# The Effect of Pre-Annealing on the Evolution of the Microstructure and Mechanical Behavior of Aluminum Processed by a Novel SPD Method

**DOI:** 10.3390/ma13102361

**Published:** 2020-05-21

**Authors:** Alexander P. Zhilyaev, Mario J. Torres, Homero D. Cadena, Sandra L. Rodriguez, Jessica Calvo, José-María Cabrera

**Affiliations:** 1 Laboratory of Mechanics of Gradient Nanomaterials, Nosov Magnitogorsk State Technical University, 455000 Magnitogorsk, Russia; alexz@anrb.ru; 2 Institute for Metals Superplasticity Problems, 450001 Ufa, Russia; 3 Department of Materials Science and Engineering, EEBE—Universitat Politècnica de Catalunya, 08019 Barcelona, Spain; mxtm45@hotmail.com (M.J.T.); cadenahd@gmail.com (H.D.C.); jessica.calvo@upc.edu (J.C.); 4 Department of Mechanical Engineering, Faculty of Engineering, Autonomous University of San Luis Potosi, San Luis Potosi 78290, Mexico; sandyreyna@uaslp.mx; 5 Institute of Metallurgical and Material Research, Universidad Michoacana de San Nicolás de Hidalgo, Morelia, Michoacan 58230, Mexico

**Keywords:** CCDF, UFG, EBSD, mechanical properties, pre-annealing, aluminum

## Abstract

A novel continuous process of severe plastic deformation (SPD) named continuous close die forging (CCDF) is presented. The CCDF process combines all favorite advances of multidirectional forging and other SPD methods, and it can be easily scaled up for industrial use. Keeping constant both the cross section and the length of the sample, the new method promotes a refinement of the microstructure. The grain refinement and mechanical properties of commercially pure aluminum (AA1050) were studied as a function of the number of CCDF repetitive passes and the previous conditioning heat treatment. In particular, two different pre-annealing treatments were applied. The first one consisted of a reheating to 623 K (350 °C) for 1 h aimed at eliminating the effect of the deformation applied during the bar extrusion. The second pre-annealing consisted on a reheating to 903 K (630 °C) for 48 h plus cooling down to 573 K (300 °C) at 66 K/h. At this latter temperature, the material remained for 3 h prior to a final cooling to room temperature within the furnace, i.e., slow cooling rate. This treatment aimed at increasing the elongation and formability of the material. No visible cracking was detected in the workpiece of AA1050 processed up to 16 passes at room temperature after the first conditioning heat treatment, and 24 passes were able to be applied when the material was subjected to the second heat treatment. After processing through 16 passes for the low temperature pre-annealed samples, the microstructure was refined down to a mean grain size of 0.82 µm and the grain size was further reduced to 0.72 µm after 24 passes, applied after the high temperature heat treatment. Tensile tests showed the best mechanical properties after the high temperature pre-annealing and 24 passes of the novel CCDF method. A yield strength and ultimate tensile strength of 180 and 226 MPa, respectively, were obtained. Elongation to fracture was 18%. The microstructure and grain boundary nature are discussed in relation to the mechanical properties attained by the current ultrafine-grained (UFG) AA1050 processed by this new method.

## 1. Introduction

It is well known that the microstructure plays an important role in the physical and mechanical properties of polycrystalline materials. According to the Hall–Petch relationship, which describes the dependence between the yield stress, *σ_y_*, and the grain size, *d*, in Equation (1) [1,2], the strength of metallic materials can be enhanced by grain refinement. During the last three decades, severe plastic deformation (SPD) methods have been successfully applied to achieve ultrafine-grained (UFG) materials, in the range between 100 nm and 1 µm, or even nanoscale structure in numerous pure metals and alloys [3,4]. Almost 20 years ago, the intention for a transfer of SPD to industry was disclosed [5,6]. However, no significant progress in that direction has occurred.

(1) $$\sigma_{y}=\sigma_{0}+k\;\cdot \;d^{-1/2}$$

It is also well known that when a SPD process, performed at low temperatures, is applied to a given metallic material, a UFG structure can be achieved where most of the grain boundaries are of the high angle type. A large amount of strain ε must be applied (i.e., larger than 5–7) to be considered as a SPD process. The presence of large hydrostatic pressures in order to avoid crack appearance, which would compromise the integrity of the workpiece, is also compulsory. Currently, several SPD processes have been proposed in the literature, meeting the latter specifications and generating uniform microstructures throughout the volume of the piece. This homogeneity is particularly important to ensure the stability of the mechanical properties and subsequent forming processes.

There are basically two ways for mass production of UFG materials, namely, top-down and bottom-up techniques. While the latter is based on nano-powder compaction, the first one is preferred because the material obtained presents advantages such as lack of porosity, reduced levels of impurities, and the possibility of scaling the process industrially, in terms of both the processing times and required investment [7,8]. Although High-Pressure Torsion (HPT) and Equal Channel Angular Pressing (ECAP) are the two most popular SPD process, and many other processes are constantly being proposed [9], Multidirectional Forging (MDF) has also the advantage of being easily scalable to industrial production [3,4,5,6,10]. MDF is based on the repetitive application of compression to a metal, while varying the deformation axis after each pass. This promotes accumulation of redundant plastic deformation, either at low or high temperatures. A key factor for the microstructural refinement in this process is the repetitive variation of the axis where the deformation is applied [3,8].

In this research work, a novel MDF method [11], called Continuous Closed Die Forging (CCDF), has been applied to a commercially pure aluminum (AA1050) with 99.5% purity. The main advantage of this new process is the simplicity of the set-up, and the ability to manage large billets or samples. It is slightly different to conventional MDF processes, because the cross section here is circular or elliptical, minimizing friction problems. In addition to the new processing method, the material was subjected to two different pre-annealing treatments. The first pre-annealing treatment was performed at a low temperature for a short time, and the second one at high temperature for a long time. According to the results, the new CCDF process was suitable to generate UFG microstructures regardless of the conditioning of the samples by different pre-annealing heat treatments. However, the pre-annealing conditions affected the number of deformation passes that could be applied to the samples and, thus, the final grain size and mechanical properties.

## 2. Materials and Methods

Bars of EN AW-1050 (AA1050) with a diameter of 20 mm and a length of 100 mm were subjected to two different annealing heat treatments. These conditions were selected in order to remove the effect of any strengthening generated during the extrusion process and homogenize the microstructure. The first annealing heat treatment was performed at 623 K (350 °C) for 1 h followed by air cooling. A homogeneous and equiaxic grain size of 160 μm was achieved. The second pre-annealing consisted of a reheating to 903 K (630 °C) for 48 h, and then cooling down to 573 K (300 °C) at 66 K/h. At this latter temperature, the material was soaked for 3 h prior to a slow cooling (i.e., within the furnace) to room temperature. Under this condition, the microstructure was somehow heterogeneous, as 80% of the area was occupied by grains sizes as large as 600 μm, while the rest of the grains remained at 160 μm. The average grain size was 400 µm. This pre-annealing heat treatment was selected based on a study reported in the literature concerning a work to increase the yield strength of AA1050 processed by HPT [12,13]. A similar effect of the pre-annealing treatment in increasing the yield strength is expected for the present SPD process. The heat treatments were applied to the samples using an HOBERSAL 12 PR/300 oven (HOBERSAL, Barcelona, Spain).

**Figure 1:**
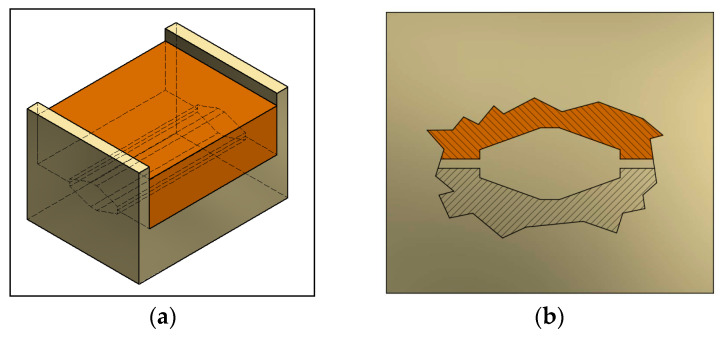

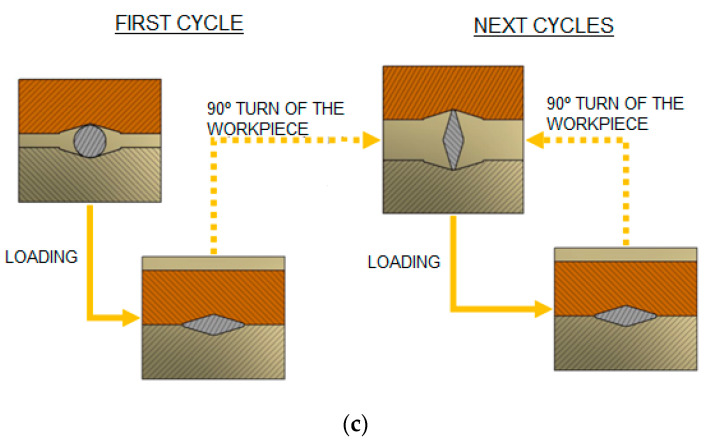
Schematic of continuous closed die forging (CCDF) including (**a**) the matrix configuration, (**b**) geometry of the dies, and (**c**) deformation sequence.

The principle of CCDF processing is shown in Figure 1. The tool (Figure 1a) is composed of two dies that form a rhomboidal inner cross section, as indicated in Figure 1b, with a relationship between the major and minor axis of 3:1. The geometry has been defined in order to avoid tilting of the samples between deformation passes. The deformation sequence is described in Figure 1c, indicating that the samples, with an initial circular section of 20 mm in diameter, were placed in the cavity of the tool, and a closing load of 431 kN was applied to the dies through a plunger, which was held for 10 s, forcing the sample to adopt the inner shape of the dies. After the plunger retraction, the dies were open and the sample turned 90 degrees counterclockwise for all the subsequent forming passes. In this new position of the sample, a closing load was again applied and the material flowed until it filled the space between the dies. The final geometry of the samples after each deformation pass was rhomboidal with a major axis of less than 15 mm and a second axis that depended on the hardness of the material, i.e., on the number of passes. Molybdenum disulfide was used as lubricant in the process. The plunger speed was 5 mm s^−1^ and the temperature was controlled using a thermocouple and a data acquisition system. The maximum increment in temperature measured under the deformation conditions was only 4 K, so the process could be assumed to be isothermal. Following this CCDF route, up to 16 passes could be applied to the material that followed the low-temperature pre-annealing condition and a total of 24 passes could be applied to the AA1050 samples that followed the high temperature pre-annealing. These were the maximum number of passes before crack appearance. It is worth mentioning that the length of the samples was kept constant during all passes.

For metallographic analysis, sectioned samples extracted from the central part of the cross section were electropolished and the grain structures were studied by orientation imaging microscopy (OIM) using the electron back scattered diffraction (EBSD,) technique integrated in a Scanning Electron Microscope (SEM) brand JEOL (Tokyo, Japan), model JSM-5600 (controlled and analyzed using Channel 5 software 2010). The grain size measurement was slightly different between the samples, and therefore appropriate area dimensions and step sizes (no lower than 0.03 μm) were chosen to maximize the number of data points and ensure a good statistical results. In the case of a few grains per picture, several images were taken. The statistical variations in grain size and misorientation angle, obtained by EBSD, were correlated with the mechanical results. Additionally, the mechanical properties were determined on cylindrical mini samples machined from the bars after CCDF processing using a universal machine INSTRON model 4507 (INSTRON, Norwood, MA, USA). Tensile samples were extracted in the longitudinal direction from the central part of the cross section with the following dimensions: length: 45 mm; diameter: 5 mm; and gage length: 20 mm. They were machined by a wire cutting process. The latter tests were carried out until the rupture of the sample and only on specimens with the maximum number of CCDF passes. Tests were performed at an initial strain rate of 0.001 s^−1^ using a pre-load of 10 N.

## 3. Results and Discussion

### 3.1. Effect of the Pre-Annealing Cycle on the Microstructure after 8 Passes

**Figure 2:**
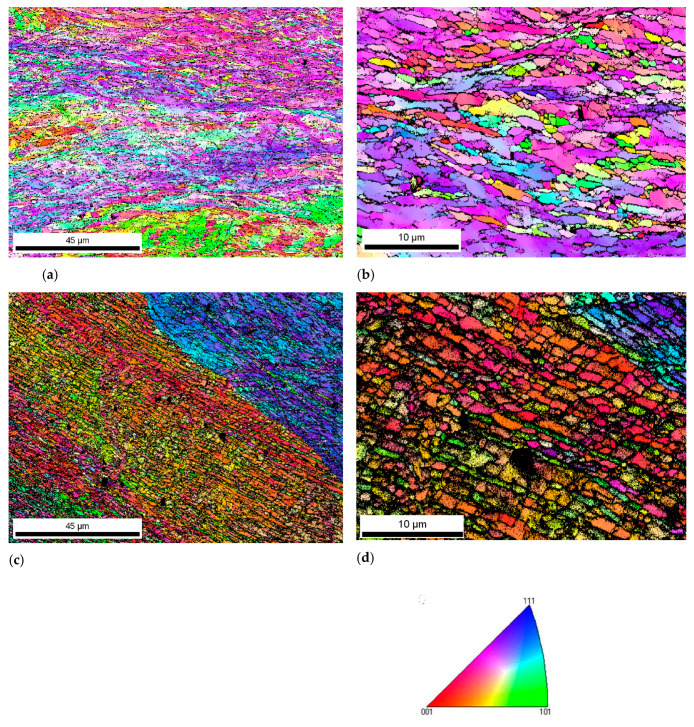
Electron back scattered diffraction (EBSD) results after eight passes of CCDF: texture maps of (**a**,**b**) AA1050 pre-annealed at low temperature and (**c**,**d**) AA1050 after pre-annealing at high temperature.

The microstructure generated in the cross sectional area, after the same number of passes, in this case eight passes, was compared for a sample that had followed the pre-annealing at low temperature and a sample that had followed a pre-annealing at high temperature. Figure 2 represents the EBSD texture maps for both samples. In both cases, the material has undergone a refinement of the microstructure with the formation of small grains and subgrains, which exhibit a preferable orientation related to the orientation of the original grain generated during the pre-annealing treatment. It is worth mentioning that a Finite Element simulation [14] had been carried out to evaluate the magnitude and distribution of the strain applied after each deformation pass. Results indicated that there is some heterogeneity in the cross sectional area of the samples. While the central part attained a total strain of 7 after 8 CCDF passes (and consequently, 14 after 16 passes and 21 after 24 passes), the strain in the outer region of the cross section achieved a strain of 3. The microstructural and mechanical study was always done on the center region of the cross section, where the strain was maximum.

**Figure 3:**
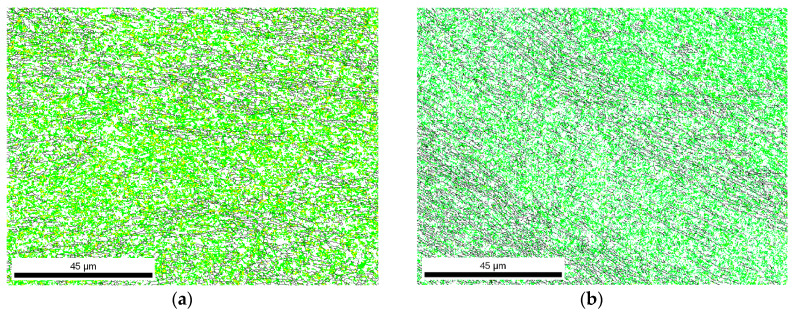

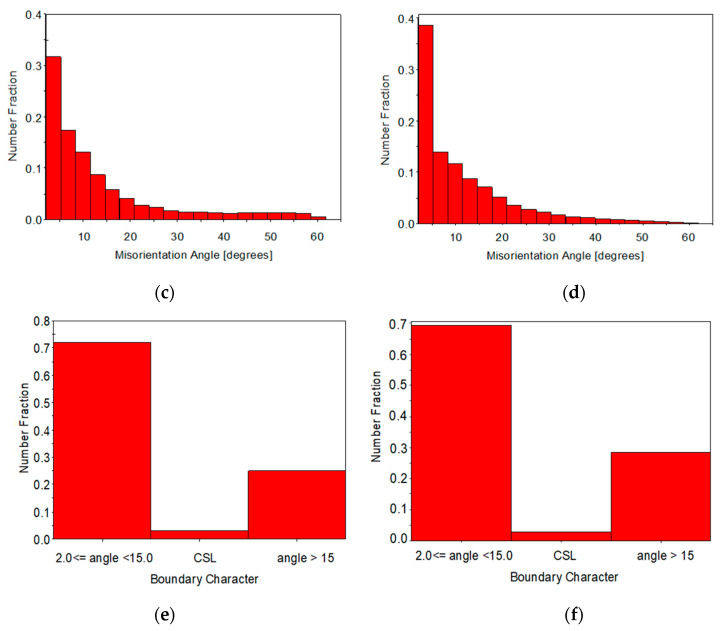
Grain boundary maps of AA1050 after eight passes with a pre-annealing at (**a**) low temperature and (**b**) high temperature, statistical analysis of boundaries misorientation for the samples with pre-annealing at (**c**) low temperature and (**d**) high temperature, and quantitative analysis of Low Angle Boundaries (LABs) and High Angle Boundaries (HABs) for a pre-annealing at (**e**) low temperature and (**f**) high temperature.

If EBSD data is analyzed in more detail, and the orientation of the grain boundaries is taken into account, small differences are detected for the different pre-annealing conditions. Figure 3a,b represents the grain boundary maps for the low and high temperature pre-annealing, respectively. In this case, HABs (High Angle Boundaries, i.e., misorientation angles larger than 15°) are represented as black lines, while the LABs (Low Angle Boundaries, i.e., misorientation angles lower than 15°) are represented as green lines. In both cases, LABs are more common than HABs. The statistical analysis of the fraction of boundaries according to their misorientation is represented in Figure 3c,d for the low- and high-temperature annealing, respectively. In this case, the fraction of LABs with the smallest misorientation represented in the graph is slighter higher for the sample pre-annealed at high temperature. However, when the boundaries are divided between those with a misorientation lower than 15° and the ones with a misorientation higher than 15° (Figure 3e,f, for the low-temperature pre-annealing and high temperature pre-annealing, respectively), the results show that the high temperature annealing promotes more HABs, and thus newer grains, than the low temperatures pre-annealing, which is related to a higher fraction of subgrains.

**Figure 4:**
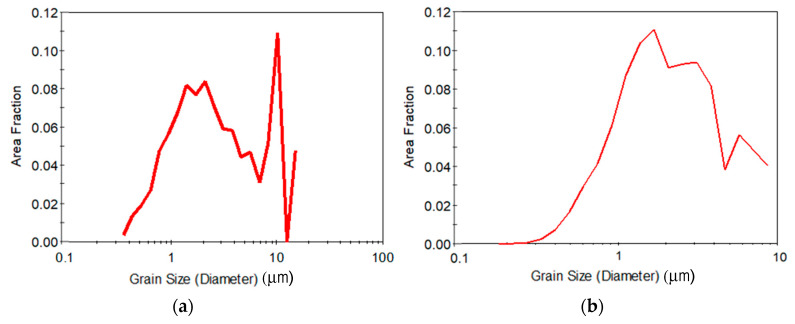
Distribution of the grain size after eight passes for (**a**) the sample pre-annealed at low temperature and (**b**) the sample pre-annealed at high temperature.

Finally, the distribution of the grain size is represented in Figure 4 for both pre-annealing conditions. The distribution of the grain size is clearly log-normal and more homogeneous for the high temperature pre-annealing condition, although the distribution does not correspond to a UFG material for any of the cases, indicating that eight passes is not enough, regardless of the pre-annealing treatment, to promote the desired refined microstructure for the current new CCDF process.

### 3.2. Characterization of the Microstructure at Maximum Number Passes

**Figure 5:**
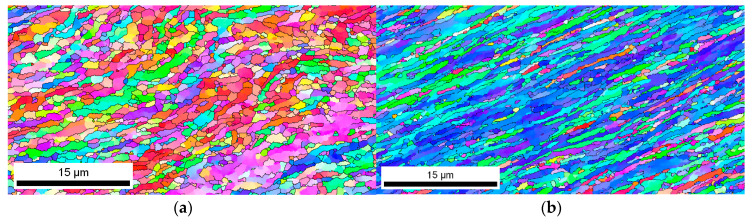
EBSD texture maps for (**a**) sample subjected to a low temperature pre-annealing after 16 CCDF passes and (**b**) sample subjected to a high temperature pre-annealing after 24 CCDF passes.

The maximum number of passes that could be applied to the material, through the novel CCDF process, was 16 for the material pre-annealed at low temperature and 24 for the material pre-annealed at high temperature. Therefore, the first difference between the two pre-annealing treatments was the number of passes and accumulation of the deformation that could be applied, which were higher for the high temperature pre-annealing. Figure 5 represents the EBSD texture maps at the maximum number of passes for each pre-annealing condition. In both cases, the samples contain a mixture of elongated and equiaxed grains.

**Figure 6:**
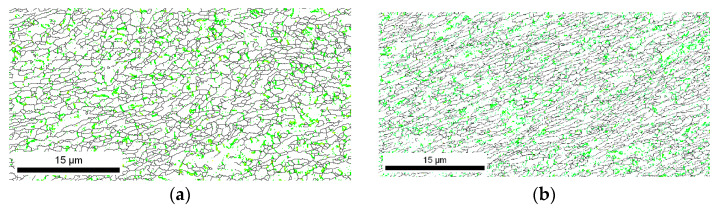

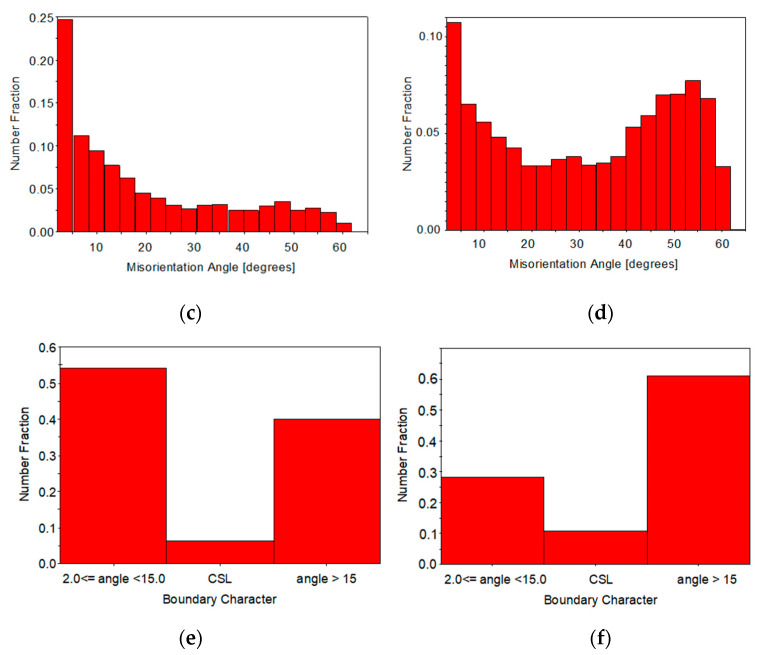
Grain boundary maps of Al 1050 after the maximum number of passes with a pre-annealing at (**a**) low temperature and (**b**) high temperature, statistical analysis of boundaries misorientation for the samples with pre-annealing at (**c**) low temperature and (**d**) high temperature, and quantitative analysis of LABs and HABs for a pre-annealing at (**e**) low temperature and (**f**) high temperature.

If the characteristics of the grain boundaries are analyzed (Figure 6), the increase in the fraction of HABs with respect to the microstructures after eight passes is evident (Figure 3). However, there is still a high fraction of LABs that is significantly higher after 16 deformation passes than after 24 deformation passes. Therefore, the possibility of increasing the amount of deformation that can be accumulated by increasing the pre-annealing temperature and time promotes the formation of new grains and reduces the number of subgrains. The misorientation distribution of boundaries obtained from the EBSD data and displayed in Figure 6c,d correspond to a bimodal type with two clear different populations—low and high angles—as usually reported in the literature for highly deformed samples [15,16,17,18,19,20]. Present results are also in agreement with Chang et al.’s observations [21]. These authors reported that the dislocation density inside the grains of an AA1050 alloy decreases at increasing strain, while most of the grains eventually became free of dislocations, due to a continuous dynamic recrystallization during the SPD.

**Figure 7:**
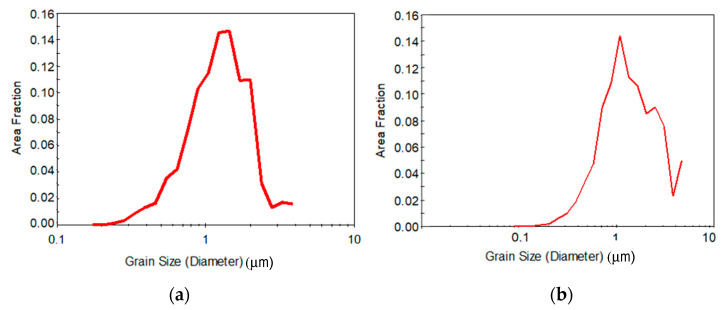
Distribution of the grain size after the maximum number of passes with a pre-annealing at (**a**) low temperature and (**b**) high temperature.

If the effect of pre-annealing on the grain size is taken into account, as represented in Figure 7, both pre-annealing conditions promote distributions that correspond to UFG materials, where the log-normal distribution is centered in UFG sizes and the distribution shows a clear homogeneity. The EBSD data reveal that the average grain size has been reduced to 0.82 µm after 16 passes of CCDF for a sample pre-annealed at low temperature and to 0.72 µm after 24 passes of CCDF for a sample pre-annealed at high temperature for a long time. According to these results, it can be stated that the CCDF process that has been proposed in this work as a novel SPD alternative can effectively promote UFG microstructures.

### 3.3. Evolution of the Mechanical Properties

**Figure 8:**
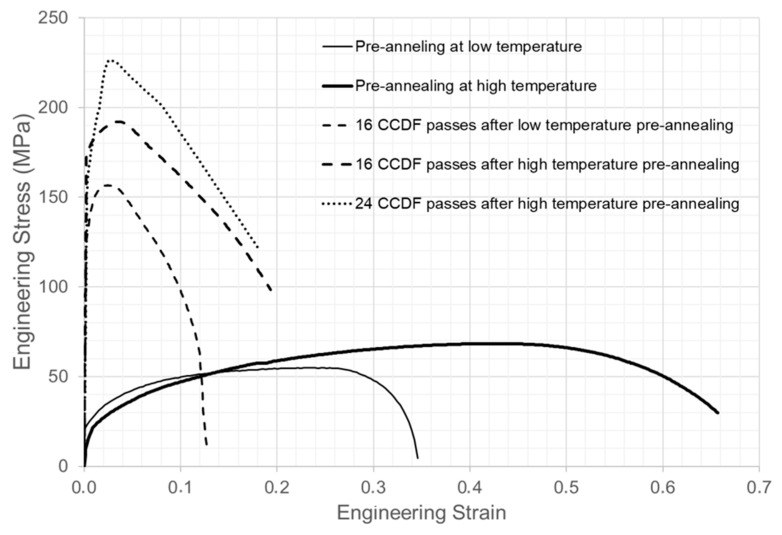
Stress-strain curves of AA1050 after different pre-annealing and CCDF passes.

Figure 8 represents the stress-strain curves of the samples subjected to different pre-annealing treatment and deformation passes. When the curves corresponding to the pre-annealing at low and high temperatures are compared, it is evident how increasing the temperature and time of the pre-annealing promotes a curve with a higher ultimate tensile strength (UTS) and the elongation is almost doubled. This increase in the elongation and ductility of the material could explain the reason why the number of the deformation passes could be increased after the pre-annealing at high temperature.

After 16 CCDF passes, the microstructure exhibited the characteristics of UFG materials (Figure 5 and Figure 7), and the stress-strain curves in Figure 8 show that the refinement of this microstructure can be related to an increase of the yield stress (YS) and UTS and a reduction of the elongation, regardless of the pre-annealing treatment. In fact, the uniform elongation (elongation up to the UTS) is severely reduced but the material exhibits high non-uniform elongation, indicating that it is able to absorb energy during its plastic deformation. If the effect of the pre-annealing treatment in the stress-strain behavior of the material after 16 CCDF passes is analyzed, it can be inferred that the high temperature pre-annealing promotes higher UTS and elongation values and it is more effective when trying to optimize the mechanical properties promoted by the novel CCDF process. Therefore, pre-annealing at high temperatures not only promotes better mechanical properties for a constant number of deformation passes, but it also increases the formability, allowing one to increase the total number of deformation passes that can be applied. This increase in the number of deformation passes promotes an extra increase of the UTS with small effect on the elongation to rupture.

In general terms, if pre-annealing at low temperature allows the application of 16 CCDF passes, related to a YS of 122 MPa, a UTS of 156 MPa and 12% of elongation to rupture, these properties can be increased to a YS of 180 MPa, UTS of 226 MPa, and 18% elongation to rupture if the material is pre-annealed at high temperature and time and subjected to 24 deformation passes. The average increase of the mechanical properties is in excess of 40%. This confirms the results of previous works that indicate that an initial heat treatment prior to deformation at a relatively high temperature has a positive significant effect on the mechanical behavior of the material after SPD [11]. This condition is attributed to the ability of the material to withstand high deformations.

## 4. Conclusions

Through a novel method of severe plastic deformation, called CCDF, an ultrafine grain size was obtained, which validates the concept. The material that has been used is an AA1050 alloy subjected to different pre-annealing conditions. The evolution of the microstructure is similar in the first deformation passes for which the material is not completely refined (a large substructure is still notice), but the processing window and mechanical properties are very sensitive to the initial conditioning of the material. When the material is pre-annealed a 623 K (350 °C) for 1 h, the maximum number of deformation passes is limited to 16; the average grain size is 0.82 µm; and the mechanical properties are 122 MPa of YS, 156 MPa of UTS, and 12% elongation to ruptures. These properties are in accordance with UFG materials, but they can be further improved if the material is subjected to pre-annealing at 903 K (630 °C) for 48 h followed by a cooling to 573 K (300 °C), the temperature at which the material remained for 3 h prior to a final furnace cooling to room temperature. In this case, the total number of deformation passes could be increased to 24, promoting finer average grain size of 0.72 µm, and the mechanical properties were increased by an extra 40%. This condition is attributed to the ability of the material to withstand high deformations.
